# Clinical Ramifications of Bacterial Aggregation in Pleural Fluid

**DOI:** 10.3390/idr16040046

**Published:** 2024-07-18

**Authors:** James B. Doub, Nicole Putnam

**Affiliations:** 1 The Doub Laboratory of Translational Bacterial Research, University of Maryland School of Medicine, Baltimore, MD 21201, USA; 2 Division of Clinical Care and Research, Institute of Human Virology, University of Maryland School of Medicine, Baltimore, MD 21201, USA; 3 Department of Pathology, University of Maryland School of Medicine, Baltimore, MD 21201, USA

**Keywords:** bacterial aggregation, antibiotic resistance, empyema, tissue plasminogen activator, *Staphylococcus aureus*

## Abstract

*Background:* Bacterial aggregation has been well described to occur in synovial fluid, but it is unknown if bacteria form aggregates in body fluids beyond the synovial fluid. Consequently, this translational study evaluated the ability to form bacterial aggregates in different pleural fluids. *Methods:* Four of the most common causes of thoracic empyema—*Streptococcus mitis*, *Streptococcus pneumoniae*, *Staphylococcus aureus*, and *Pseudomonas aeruginosa*—were used here. The different pleural fluids included one transudative and two exudative pleural fluids. Twenty-four-well microwell plates were used to form the aggregates with the aid of an incubating shaker at different dynamic conditions (120 RPM, 30 RPM, and static). The aggregates were then visualized with SEM and evaluated for antibiotic resistance and the ability of tissue plasminogen activator (TPA) to dissolve the aggregates. Statistical comparisons were made between the different groups. *Results:* Bacterial aggregates formed at high shaking speeds in all pleural fluid types, but no aggregates were seen in TSB. When a low shaking speed (30 RPM) was used, only exudative pleural fluid with a high protein content formed aggregates. No aggregates formed under static conditions. Furthermore, there was a statistical difference in the CFU/mL of bacteria present after antibiotics were administered compared to bacteria with no antibiotics (*p* < 0.005) and when TPA plus antibiotics were administered compared to antibiotics alone (*p* < 0.005). *Conclusions:* This study shows that bacteria can form aggregates in pleural fluid and at dynamic conditions similar to those seen in vivo with thoracic empyema. Importantly, this study provides a pathophysiological underpinning for the reason why antibiotics alone have a limited utility in treating empyema.

## 1. Introduction

Bacterial aggregation has been well described to occur in synovial fluid, in which aggregates harbor bacteria that are resistant to high concentrations of antibiotics as well as bacteriophages [1,2,3,4]. The mechanism behind bacterial aggregation is centered around microbes interacting with in vivo polymers, such as fibrinogen, to form aggregates [1,5]. Thus, agents that disrupt fibrin clots, like tissue plasminogen activator (TPA), have been shown to disperse the aggregates, thereby making the microbes vulnerable to antibiotics [1,2,3]. However, there is a paucity of data describing the ability of bacteria to form aggregates in body fluids beyond synovial fluid.

Consequently, it is vital to understand the ability of bacteria to form aggregates in protein-rich pleural fluid. This is because thoracic empyema is a devastating infection of the pleural space, and these infections occur in exudative pleural fluid with high protein concentrations. The management of thoracic empyema, at minimum, requires drainage with chest tubes and antibiotics. The use of TPA and DNAse, instilled through chest tubes, is also commonly used to disrupt adhesions in the pleural space, thereby allowing for adequate chest tube drainage [6]. The use of TPA and DNAse has been evaluated in several studies, which have shown clinical efficacy of these agents in the treatment of thoracic empyema [7,8]. Yet, it is unknown if these agents aid in disrupting pleural fluid bacterial aggregates. Therefore, the aim of this translational study was to evaluate the proclivity of bacteria to form aggregates in pleural fluid as well as the clinical ramifications of bacterial aggregation in pleural fluid with respect to antibiotic resistance and the ability of TPA to help dissolve the aggregates.

## 2. Materials and Methods

### 2.1. Pleural Fluid and Bacteria

This study was approved by the University of Maryland Internal Review Board (HP-00105949). Care was taken to use pleural fluid that was sterile, as proven by negative cultures of antibiotics. Three types of pleural fluid were obtained, which included transudate, exudate with low protein content (met Light’s criteria based on elevated pleural LDH), and exudate with high protein composition (met Light’s criteria based on elevated pleural LDH and protein) [9]. These three different pleural fluids were chosen to replicate normal homeostatic pleural fluid (transudate) and pleural fluid in diseased states (exudate with either high or low protein content). The bacteria used in this study were obtained from historic empyema cases that had been preserved at −80 °C, including *Staphylococcus aureus*, *Streptococcus pneumoniae*, *Streptococcus mitis*, and *Pseudomonas aeruginosa*. 

### 2.2. Bacterial Aggregation in Pleural Fluid under Different Conditions and Visualization of Aggregates

Bacterial isolates were first grown in tryptic soy broth (TSB) at 37 °C for 16 h. Then, 100 µL of bacterial growth was added to 24-well microwell plates (Corning Inc., Kennebunk, NY, USA) with either 1 mL of TSB or 1 mL of pleural fluid. The microwell plates were then incubated at 37 °C for 24 h with either static or dynamic incubation (30 RPM or 120 RPM) by placing the microwell plates on an incubating shaker (New Brunswick Scientific, Edison, NJ, USA). Aggregates that formed were imaged using an iPhone(Version 8, Apple Inc., Cupertino, CA, USA) camera and then were fixed in 2% paraformaldehyde and 2.5% glutaraldehyde. They were then dehydrated and imaged using scanning electron microscopy (SEM; FEI Quanta 200, Hillsboro, OR, USA).

### 2.3. Quantifying Bacteria Concentrations before and after Tissue Plasminogen Activator and Antibiotics

A commonly used tissue plasminogen activator (TPA) (Alteplase, Genetech, San Francisco, CA, USA) was used in this experiment. The TPA was used at a final concentration of 2 mg/2.2 mL, which is a dose commonly used clinically. The antibiotics used in this experiment were vancomycin and gentamicin. Vancomycin was used for *S. aureus*, *S. pneumoniae*, and *S. mitis*. Vancomycin stock solution was diluted to create concentrations of 100 µg/mL. For *P. aeruginosa*, gentamicin stock solution was used. This was diluted to create concentrations of 250 µg/mL.

To conduct this experiment, aggregates were allowed to form in microwell plates at low shaking (30 RPM) in exudative pleural fluid for 24 h, as previously discussed. Concentrations of bacteria were measured in colony-forming units (CFU/mL) by adding 200 µg/mL of proteinase K (Sigma Aldrich, St. Louis, MI, USA) to the wells, serially diluting samples, and plating on TSA plates for quantification. This was repeated for wells that had only antibiotics, only TPA, a combination of antibiotics and TPA, and no intervention (negative control). Plates were then incubated for an additional 16 h prior to quantification by counting CFU/mL. Bacteria were also grown in TSB and were assessed for CFU/mL after being exposed to the same agents as were used with pleural fluid. Using an unpaired *t*-test, the differences in CFU/mL were analyzed between the groups for each bacterial species. For comparisons, *p* < 0.05 was considered statistically significant. All experiments were conducted in triplicate and repeated twice. 

## 3. Results

**Figure 1:**
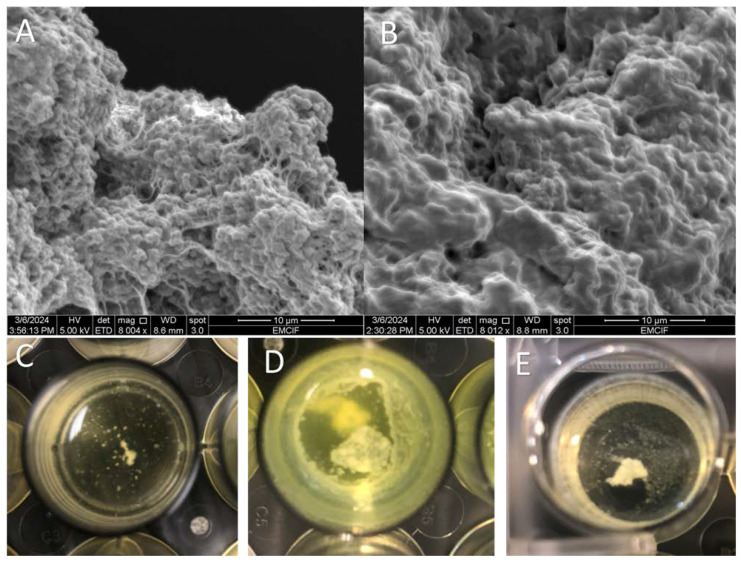
Scanning electron microscopy (SEM) and gross images of pleural fluid aggregates. (**A**) SEM of *S. aureus* pleural fluid aggregate at 8000× magnification. (**B**) SEM of *P. aeruginosa* pleural fluid aggregate at 8000× magnification, in which mucoid coat surrounds the aggregate. Gross images of (**C**) *S. mitis*, (**D**) *P. aeruginosa*, and (**E**) *S. aureus* pleural fluid aggregates.

Bacterial aggregation was observed to occur in all pleural fluids when a high shaking speed (120 RPM) was used, but not in TSB. All four bacterial species evaluated formed aggregates at the high shaking speed (Table 1; Figure 1A–E). However, when a low shaking speed (30 RPM) was used, only bacteria grown in exudative pleural fluid with a high protein content, as evaluated by Lights criteria, formed visible aggregates. In the SEM images, we observed pleural fluid aggregates of dense collections of bacteria with polymers (Figure 1A,B). Notably, *P. aeruginosa* also produced a thick mucinous layer in the exudative pleural fluid microwells (Figure 1B,D). 

**Figure 2:**
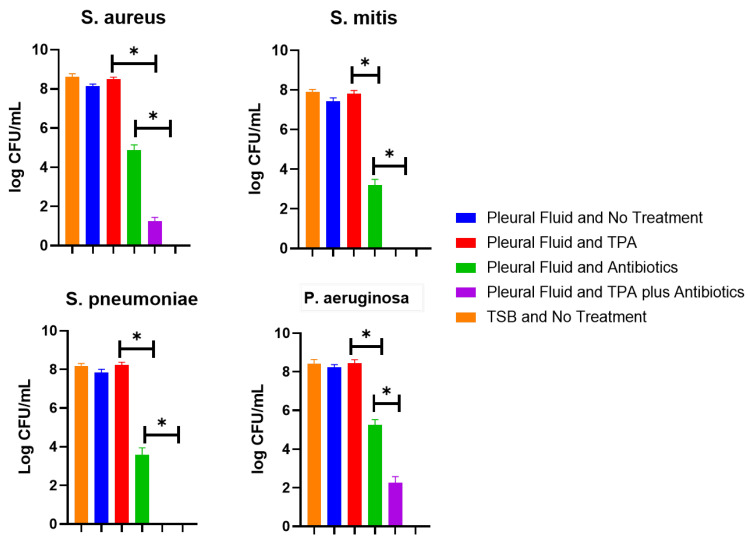
Effect of tissue plasminogen activator on pleural fluid aggregates. All four bacterial species were grown in exudative pleural fluid and allowed to form aggregates. The aggregates were then treated with antibiotics, TPA, or a combination of TPA and antibiotics, and comparisons of CFU/mL for all groups were conducted. All bacteria had no observable CFU/mL when antibiotics were added to TSB. Furthermore, all bacteria in pleural fluid had statistically significant (* *p* < 0.005) reductions in CFU/mL when antibiotics were added. When TPA was combined with antibiotics, there was a statistically significant reduction in CFU/mL (* *p* < 0.005) compared to antibiotics alone or with no treatment. However, for *P. aeruginosa* and *S. aureus*, there were residual bacteria present, but for *S. mitis* and *S. pneumoniae*, there were no detectable bacteria present.

When we evaluated the ability of TPA to disperse these aggregates, all aggregates were dispersed by TPA, but no statistical differences in CFU/mL were observed when compared to wells with no treatment (Figure 2). When antibiotics were added alone to the wells, there was a statistically significant reduction (*p* < 0.005) in bacteria recovered when compared to that of no treatment for all four bacterial species, but antibiotics could not eradicate all bacteria. Interestingly, a statistically significant (*p* < 0.005) synergistic effect occurred when aggregates were treated with TPA in addition to antibiotics. This was seen for all bacteria; notably, for *S. pneumoniae* and *S. mitis*, no residual bacteria could be recovered. Yet, residual viable bacteria were seen with *S. aureus* and *P. aeruginosa*. In TSB, the antibiotics completely inhibited all four bacteria.

## 4. Discussion

Thoracic empyema is a devastating infection causing significant morbidity and mortality, but little is known about the interaction of bacteria with in vivo pleural fluid polymers that are present in exudative pleural fluids. Here, we show that bacteria aggregation occurs in pleural fluid under dynamic conditions (Figure 1C–E), likely by mechanisms that have been previously proposed [10,11]. As seen on SEM, these aggregates were dense collections of bacteria and polymers (Figure 1A,B). The aggregates appear similar to those that have been widely studied in synovial fluid [12]. However, the aggregates that formed with *P. aeruginosa* were associated with the production of a significant amount of mucoid substances (Figure 1B,D), which has not been observed with synovial fluid aggregates [1,2,3,12]. 

While there were statistically significant decreases in CFU/mL after the administration of antibiotics alone (Figure 2), residual bacteria were still present, indicating that not all bacteria were incorporated in the aggregates, but bacteria that did aggregate were resistant to high concentrations of antibiotics. The ability of bacteria in aggregates to be resistant to high concentrations of antibiotics has been extensively evaluated in synovial fluid aggregates, and our findings here support the clinical relevance of pleural fluid aggregates [1,2,3]. 

However, the formation of pleural fluid aggregates was not universal with respect to different pleural fluid types and at different dynamic conditions (Table 1). All three pleural fluids formed aggregates at the high shaking speed, similar to that which occurs in the synovial fluid of ambulatory patients with prosthetic joint infections (120 RPM), reinforcing that aggregation can occur in body fluids that have high repetitive movements by which fibrinogen forces bacteria into aggregates even when low protein contents are present [10,11]. Yet, at the lower shaking speed (30 RPM), which mimicked the physiological conditions for pleural fluid movement, only exudative pleural fluid with a high protein content caused aggregate formation. This is important because the movement of pleural fluid in vivo is not similar to that of synovial fluid in a joint; rather, pleural fluid movements are suspected to be primarily influenced by respirations [13]. Thus, in pleural fluid with a low protein content, bacterial aggregation likely does not readily occur. This is likely why early parapneumonic pleural effusions can be treated with antibiotics alone, but once the protein content of pleural fluid changes to have large amounts of protein, as seen with empyema, antibiotics need to be used in combination with chest tube drainage and/or surgical interventions [14,15,16]. 

While the ability of bacteria to interact with in vivo polymers in body fluids is important to comprehend, elucidating agents that can dissolve these aggregates is of paramount importance. This is needed to provide potential therapeutic options to eradicate these aggregates. Here, we show that TPA readily dissolved these aggregates, but TPA had no activity on the bacteria, as seen in Figure 2. Yet, when antibiotics were combined with TPA, there was a statistically significant reduction in the amount of CFU/mL compared to when only antibiotics were used (Figure 2). Overall, the findings support what is seen clinically in that antibiotics alone rarely can cure thoracic empyema, given that bacteria likely interact with in vivo polymers, causing aggregates that antibiotics are not able to fully eradicate by themselves [14,15,16]. Rather, at a minimum, chest tube drainage often with the administration of TPA is required to not only degrade adhesions, allowing for enhanced drainage, but also, as seen here, to dissolve bacteria aggregates, thus allowing for systemic antibiotics to eradicate the bacteria dispersed by TPA. 

Yet, not all bacteria are the same with respect to their propensity to interact with in vivo polymers such as fibrinogen. Here, we show that for *S. mitis* and *S. pneumoniae*, there were no observable CFU/mL after the combination of TPA and antibiotics. On the other hand, for *S. aureus* and *P. aeruginosa*, there were still observable bacteria present. We hypothesize that this occurred because the production of thick mucoid substance likely contributed to the recalcitrant nature of *P. aeruginosa* for which TPA had little activity, thereby causing residual bacteria to be present [17]. For *S. aureus*, the ability of this bacterium to readily utilize membrane-bound and secreted clumping factor, as well as its propensity to convert to persister and small colony variants, which are tolerant to antibiotics, likely contributed to the persistence of bacteria [11,18]. However, more research is needed to clarify these claims. 

While this study has interesting implications, it also has some limitations. For one, this study only evaluated four bacterial species. These are the most common bacterial pathogens to cause thoracic empyema, but follow-up studies should also be conducted for evaluating the less common causes of thoracic empyema [19]. This is especially important because it may be that certain bacterial strains that cause clinical infections have adapted to be more prone to form aggregates than other strains, and thus a robust evaluation of clinical isolates is needed. Secondly, we assumed that the movement of pleural fluid in vivo is similar to that which occurs on a 30 RPM shaker. However, this is an assumption, and the real in vivo movement of pleural fluid has not been fully elucidated. Lastly, the mechanism behind the resistance to antibiotics in aggregates was not evaluated here nor was the mechanism behind the improved antibiotic effectiveness after TPA administration. One plausible reason for these findings is the conversion of bacteria from planktonic states to reduced metabolic states, such as small colony variants in aggregates and the recovery of planktonic states, when aggregates are disrupted with TPA. Yet, further studies are needed to better comprehend the mechanistic reason behind why bacteria in aggregates become resistant to antibiotics and even bacteriophages. 

## 5. Conclusions

In conclusion, bacteria interact with in vivo polymers to form aggregates in dynamic pleural fluid conditions that mimic those seen in vivo. These pleural fluid aggregates are clinically relevant because they harbor bacteria that are resistant to high concentrations of antibiotics. Yet, TPA has the ability to disperse these pleural fluid aggregates, which is advantageous given that this adjuvant agent is already widely used in the management of thoracic empyema. Thus, this study provides a mechanistic underpinning for why poor outcomes occur with antibiotics alone for thoracic empyema and reinforces the need for further translational research evaluating the pathophysiology of bacteria in in vivo conditions. 

## Figures and Tables

**Table 1:** Aggregation potential of the different pleural fluids under various conditions.

	High Shaking (120 RPM)	Low Shaking (30 RPM)	Static
Tryptic soy broth	No aggregation	No aggregation	No aggregation
Transudative	Aggregation	No aggregation	No aggregation
Low-protein exudate	Aggregation	No aggregation	No aggregation
High-protein exudate	Aggregation	Aggregation	No aggregation

## Data Availability

The data generated and analyzed during the current study are available upon reasonable request from the corresponding author.
